# The Impact of CD34+ Cells on Wound Healing Outcomes in Oral and Maxillofacial Surgery: A Systematic Review of Randomized Controlled Trials

**DOI:** 10.7759/cureus.83633

**Published:** 2025-05-07

**Authors:** Tharanikumar Sivakumar, Chandrasekaran Krithika, Chitathoor Sridhar, Elaveyini U, Parthiban Manohar, Nachiappan S

**Affiliations:** 1 Oral and Maxillofacial Surgery, Meenakshi Academy of Higher Education and Research, Chennai, IND; 2 Oral Medicine and Radiology, Meenakshi Ammal Dental College, Meenakshi Academy of Higher Education and Research, Chennai, IND; 3 Internal Medicine, Meenakshi Academy of Higher Education and Research, Chennai, IND; 4 Obstetrics and Gynecology, CSI Rainy Hospital, Chennai, IND; 5 Biotechnology, Madras Veterinary College, Chennai, IND; 6 Oral Surgery, Sathyabama University Dental College and Hospital, Chennai, IND

**Keywords:** acute and chronic wounds, acute wounds, cd34, chronic wounds, healing, maxillofacial, rct, regeneration

## Abstract

Despite significant advancements in surgery and biomaterials science, delayed healing and related complications remain major challenges. Emerging studies have highlighted the promising role of CD34+ cells in promoting accelerated wound healing. This systematic review aims to comprehensively evaluate the impact of CD34+ cells on healing outcomes in oral and maxillofacial surgery. A thorough search was conducted using PubMed and the Cochrane Library (CENTRAL via Ovid), complemented by manual screening of additional articles. Only randomized controlled trials (RCTs) involving systemic or local administration of CD34+ cells were included. Healing time and complication rates were the primary outcomes assessed. The review followed the Preferred Reporting Items for Systematic reviews and Meta-Analyses (PRISMA) guidelines and employed the Cochrane Risk of Bias 2 tool for quality assessment. Three relevant RCTs met the inclusion criteria. Regardless of the assessed risk of bias, all studies supported the potential therapeutic benefits of CD34+ cell-based interventions in oral and maxillofacial surgery. However, the findings also revealed significant gaps in current knowledge within this field. Overall, CD34+ cells show strong potential in enhancing wound healing in oral and maxillofacial procedures. This review lays the groundwork for future regenerative strategies aimed at optimizing surgical outcomes in this specialized area. The application of CD34+ cells in such contexts appears to be technically justified.

## Introduction and background

Oral and maxillofacial surgery relies heavily on effective post-surgical wound healing, which is influenced by the tissues involved and their anatomical location. Additionally, the form, function, and aesthetic outcome are especially important in this region [1]. The healing process following surgery is complex and tightly regulated, consisting of four overlapping phases: hemostasis, inflammation, proliferation, and remodeling [2].

A thorough understanding of wound healing is essential. The initial hemostasis phase establishes a fibrin-rich wound matrix, and cytokines released from platelets initiate inflammation, which is mediated by neutrophils and macrophages. This phase is responsible for clearing bacteria, debris, and enzymes that may impede healing. Next, fibroblast proliferation and neovascularization occur, leading to granulation tissue formation and eventual wound closure. The final phase, remodeling, restores the wound’s histological and functional integrity. In chronic wounds, however, the healing process is disrupted at the molecular level, with impaired inflammatory responses and delayed tissue growth. CD34+ cells have been suggested to enhance proliferation and support healing in such cases [3]. The transition from the inflammatory to the proliferative phase is particularly critical, as failure at this stage can lead to chronic wounds, hypertrophic scars, or keloids [4].

Despite substantial progress in surgical techniques and biomaterials, clinical challenges such as delayed healing, infections, and insufficient tissue regeneration persist [5]. These issues highlight the need for innovative therapeutic strategies. Regenerative medicine is gaining traction, even in minor procedures, for its potential to improve outcomes [6]. Within this field, CD34+ cells have emerged as key players, often referred to as “messengers of healing” [7]. Unlike simple repair, regeneration offers a more effective pathway to restore function, and research into molecular signals, biomaterials, and cell-based therapies continues to expand [8].

CD34+ cells are a subset of progenitor cells found predominantly in bone marrow, peripheral blood, and umbilical cord blood [9]. They are identified by the surface expression of CD34, a glycoprotein involved in cell adhesion and migration. These cells possess multipotent regenerative abilities and have been shown to support tissue repair through neovascularization, immune modulation, and remodeling [10]. Their efficacy has been demonstrated in enhancing wound healing in fields such as cardiology [11], dermatology [12], and orthopedics [13].

In the context of oral and maxillofacial surgery, CD34+ cells show particular promise due to their angiogenic potential. By secreting bioactive molecules like vascular endothelial growth factor, basic fibroblast growth factor, and interleukins, they can promote the proliferation of fibroblasts and keratinocytes, thus enhancing revascularization and tissue repair [14]. Despite this promising potential, their application in oral and maxillofacial surgery remains insufficiently documented.

While several studies have explored the utility of CD34+ cells across various surgical specialties, reporting accelerated healing and reduced complication rates, their specific effects on oral and maxillofacial wound healing remain under-investigated [15]. This gap in evidence underscores the need for a systematic review to consolidate current knowledge and assess the clinical potential of CD34+ cells in this specialized domain.

Given their therapeutic promise, CD34+ cells could play a transformative role in improving healing outcomes and patient quality of life in oral and maxillofacial surgery. This systematic review aims to offer a comprehensive analysis of their impact and pave the way for more widespread clinical application in the future.

## Review

Materials and methods

The systematic review was registered in PROSPERO (CRD420250654024). The objective was to identify, assess, and synthesize all randomized controlled trials (RCTs) involving patients undergoing surgical procedures related to bone, including grafting. The studies included met the following criteria: (i) systemic administration of CD34+ cells intraoperatively or perioperatively and (ii) local administration of CD34+ cells either at the surgical site or within the graft material.

When reported, healing time was considered the primary outcome. If data on complications were available, these were regarded as secondary outcomes.

PICO Information

Participants included patients undergoing bone-related surgical procedures, such as grafting. The interventions assessed were the intraoperative or perioperative administration of CD34+ cells, either systemically or locally at the surgical site, or the use of CD34+ cells incorporated into graft material. Comparator groups consisted of patients who either did not receive CD34+ cells or received a lower dose. The primary outcome was healing time, while the incidence of complications, when reported, served as secondary outcomes.

Study Design

Eligible study designs included RCTs. Searches were conducted in PubMed and the Cochrane Library (CENTRAL via Ovid), supplemented by manual searches of additional articles and websites. The search spanned from 1987 to December 4, 2024.

Search strings included (1) CD34 cells fracture healing and (2) CD34 cells oral surgery. Articles were independently screened by title and abstract by all authors. Full texts were retrieved and reviewed for those meeting eligibility criteria, while abstracts were excluded after the initial screening. Subsequently, all authors participated in citation and trial registry screening. Discrepancies were resolved through consensus. The selection process was documented in detail and presented using a Preferred Reporting Items for Systematic reviews and Meta-Analyses (PRISMA) flow diagram. Study characteristics and outcome data were extracted using a standardized form. The risk of bias for each study was independently assessed by all authors using the Cochrane Risk of Bias 2 tool.

Results

**Figure 1:**
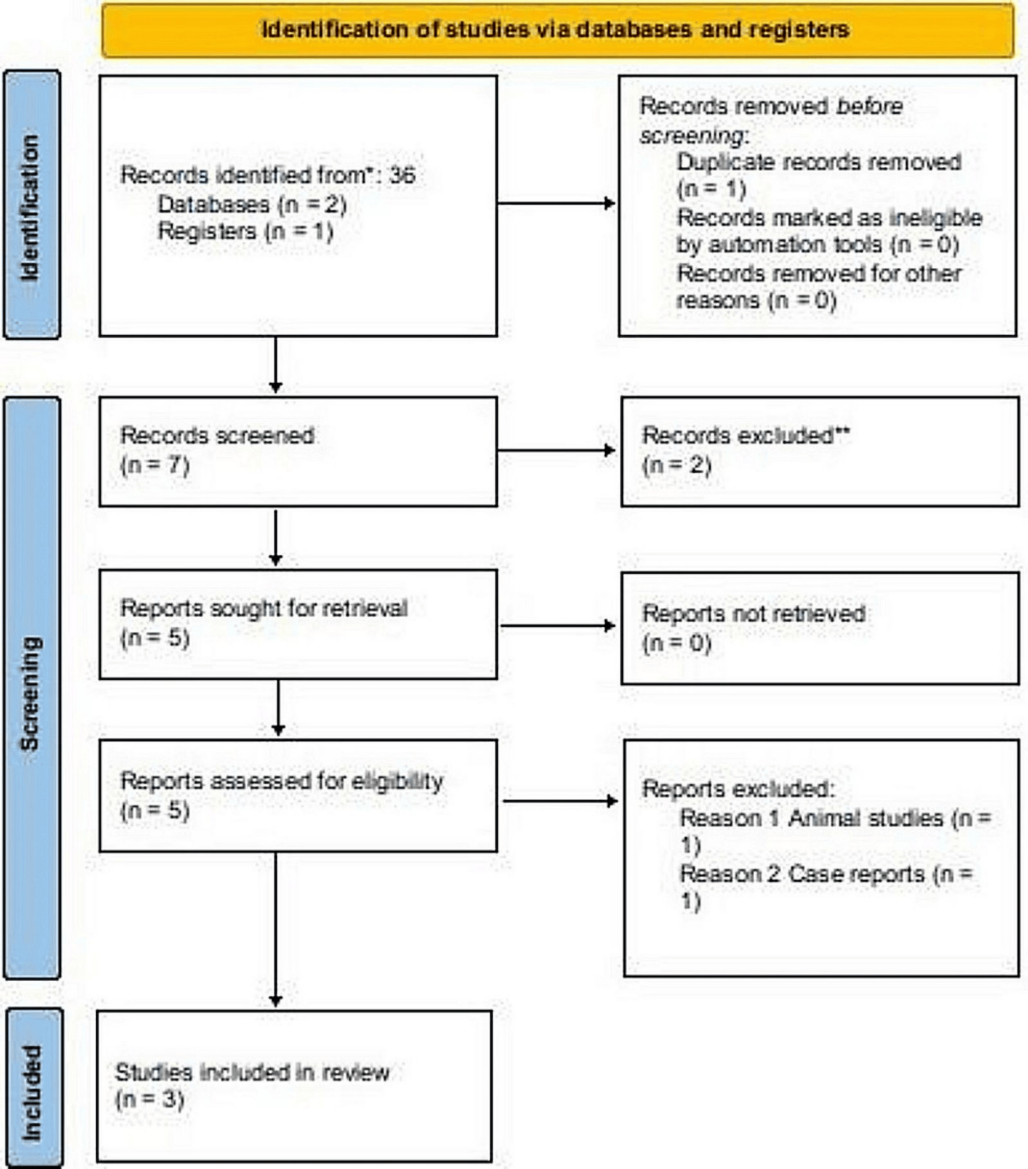
PRISMA flow diagram of the study selection process PRISMA, Preferred Reporting Items for Systematic reviews and Meta-Analyses

**Figure 2:**
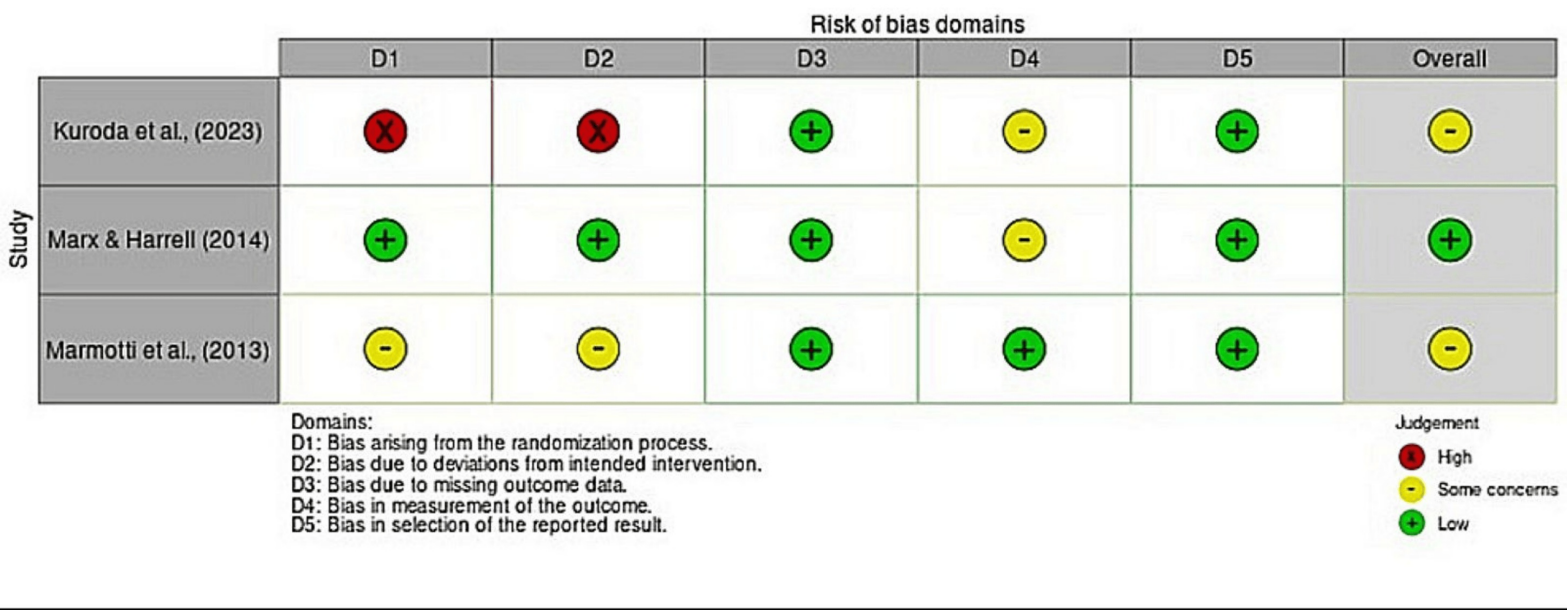
Risk of bias assessment for the included articles

The systematic search yielded 36 articles, of which three met the inclusion criteria after screening, as shown in the PRISMA flow diagram (Figure 1). Table 1 presents the included studies and highlights the potential benefits of CD34+ cells in wound healing. The risk of bias for the included studies is illustrated in Figure 2.

**Table 1:** PICO summary of the included articles G-CSF, granulocyte-colony stimulating factor

Study	Population	Intervention	Comparator	Outcome parameter	Conclusion
Kuroda et al. (2023) [16]	Fractures of tibial or femoral bones with nonunion for over six months after previous treatment, without infection, planned for autologous bone grafting, aged 20-69 years	Autologous bone graft and CD34+ cell treatment by injections	Autologous bone graft therapy	Healing time	Healing time was shorter in the intervention group.
Marx and Harrell (2014) [17]	Patients with 6- to 8-cm continuity defects in the mandible with retained proximal and distal segments	In situ tissue-engineered graft containing 1,012 ± 752 CD34+ cells/mL, 54 ± 38 CD44+, CD90+, and CD105+ cells/mL, along with rhBMP-2 in an absorbable collagen sponge (1 mg/cm of defect) and crushed cancellous allogeneic bone	In situ tissue-engineered graft containing 54 ± 38 CD34+ cells/mL, 54 ± 38 CD44+, CD90+, and CD105+ cells/mL, along with rhBMP-2 in an absorbable collagen sponge (1 mg/cm of defect) and crushed cancellous allogeneic bone	Radiographic bone density and histomorphometry	CD34+ cell counts of at least 200/mL in composite grafts are directly correlated with successful bone regeneration.
Marmotti et al. (2013) [18]	Subjects undergoing opening-wedge high tibial valgus osteotomy	Preoperative administration of G-CSF at 10 μg/kg/day for three consecutive days, with an additional half-dose four hours before the surgery	No presurgical intervention	Lysholm Knee Scale, SF-36, and radiography	G-CSF and/or mobilized bone marrow cells may accelerate bone graft substitute osseointegration.

Discussion

As mentioned in the introduction, the use of CD34+ cells in oral and maxillofacial surgery is underexplored and not well documented. This systematic review aims to gather available evidence on such procedures from indexed databases. However, the direct application of CD34+ cells in oral or maxillofacial surgery has not been reported in the literature thus far. As a result, it is necessary to examine various surgical uses of CD34+ cells to determine their potential in maxillofacial procedures.

Despite this, the findings from this systematic review highlight the potential of CD34+ cells in shifting the wound healing process toward a more favorable outcome, particularly in enhancing tissue regeneration, especially bone. While numerous reviews emphasize the potential of CD34+ cells, most evidence comes from in vitro and in vivo studies rather than clinical trials. Given the broad potential for CD34+ cells in oral and maxillofacial surgery, we reviewed clinical studies using CD34+ cells and found only three relevant studies. One study had a low risk of bias, while the other two had some concerns about their scientific rigor.

The studies predominantly underscore the contribution of CD34+ cells to improved outcomes through mechanisms like angiogenesis, osteogenesis, and modulation of inflammatory responses. Kuroda et al. (2023) [16] demonstrated that the addition of CD34+ cell treatment to autologous bone grafts significantly reduced healing time in patients with tibial or femoral bone fractures and nonunion. These findings are relevant to maxillofacial bone healing, suggesting that CD34+ cells could contribute to timely healing and improved functional and aesthetic outcomes. However, the study did not randomize participants, which raises concerns about the reliability of the results. Nonetheless, CD34+ cells were shown to assist in the healing of non-healing wounds and enhance post-surgical wound healing.

Similarly, Marx and Harrell (2014) [17] examined the effect of CD34+ cell density in composite grafts for mandibular continuity defects. They found a direct correlation between higher CD34+ cell counts and successful bone regeneration, as evidenced by increased radiographic density and favorable histomorphometric outcomes. This study provides valuable insights for surgeons, highlighting the importance of optimizing CD34+ cell concentrations to achieve enhanced bone regeneration in craniofacial defects. These results suggest that CD34+ cells also promote healing in maxillofacial regions.

Marmotti et al. (2013) [18] introduced a novel preoperative strategy using granulocyte-colony stimulating factor to mobilize bone marrow-derived CD34+ cells. Their findings showed that this approach accelerated bone graft substitute osseointegration in high tibial valgus osteotomy, as indicated by improved functional scores and radiographic outcomes. These results further demonstrate the versatility of CD34+ cells and their potential in oral and maxillofacial applications.

Together, these studies, despite varying levels of bias, emphasize the potential benefits of CD34+ cell-based therapies in oral and maxillofacial surgery. However, significant knowledge gaps remain. The unique anatomical features of the maxillofacial region, the presence of microbial flora, and the diversity of surgical techniques all warrant further investigation before CD34+ cells can be routinely used peri-surgically. Additionally, challenges such as standardizing CD34+ cell isolation and determining appropriate dosages need to be addressed [19]. Given the lack of strong evidence on the use of CD34+ cells in maxillofacial surgery, both short- and long-term studies should be conducted. Other limitations, such as regulatory issues, scalability concerns, and cost factors, also need to be considered. Therefore, future research should focus on RCTs with robust methodologies to assess both short- and long-term outcomes of using CD34+ cells.

There are no previous relevant reviews on the use of CD34+ cells in oral and maxillofacial healing, making this review one of the pioneering efforts to lay the groundwork for their use in maxillofacial surgery [20].

Limitations

Despite efforts to gather relevant data, only three articles met the inclusion criteria for this systematic review. There are several limitations to acknowledge. Many searches yielded narrative reviews, animal studies, and case reports, which were intentionally excluded due to our stringent inclusion criteria. As a result, some information may have been missed from those sources, leading to a small but acknowledged gap in the reported knowledge.

Furthermore, although the included studies provide evidence for the use of CD34+ cells in surgery, only one of them specifically addressed oral and maxillofacial procedures [17]. While the findings of the other studies are still relevant, it would have been more beneficial if all the studies had focused on maxillofacial procedures. The lack of sufficient evidence in the literature is a significant issue, and future research should prioritize maxillofacial applications. This gap in the available literature also precluded the possibility of performing a meta-analysis.

## Conclusions

While the reviewed studies present a compelling case for the therapeutic potential of CD34+ cells, further research specifically focused on oral and maxillofacial surgery is essential. This systematic review underscores the promising role of CD34+ cell therapies and lays the groundwork for advancing regenerative strategies aimed at improving healing outcomes within this specialized surgical field. The use of CD34+ cells in oral and maxillofacial surgery appears technically justified, suggesting their potential for routine intraoperative or perioperative application to promote faster and more complete healing. However, it is equally important to investigate potential side effects, as this aspect remains largely underreported in the current literature. Ultimately, this review opens a new avenue for both clinicians and researchers, offering the potential to elevate the standard of care in oral and maxillofacial surgery in the near future.
